# Innovative Fermented Beverages Made with Red Rice, Barley, and Buckwheat

**DOI:** 10.3390/foods10030613

**Published:** 2021-03-13

**Authors:** Federica Cardinali, Andrea Osimani, Vesna Milanović, Cristiana Garofalo, Lucia Aquilanti

**Affiliations:** Dipartimento di Scienze Agrarie, Alimentari, ed Ambientali (D3A), Università Politecnica delle Marche, Via Brecce Bianche, 60131 Ancona, Italy; f.cardinali@staff.univpm.it (F.C.); v.milanovic@staff.univpm.it (V.M.); c.garofalo@staff.univpm.it (C.G.); l.aquilanti@univpm.it (L.A.)

**Keywords:** red rice, barley, buckwheat, lactic acid bacteria, beverages, fermentation

## Abstract

The increase in food intolerances, allergies, and food-based lifestyle choices has dramatically increased the consumer demand for healthy foods characterized by pleasant sensory traits. In such a context, innovative cereal-based beverages are characterized by high nutritional value, pleasant palatability, and potential healthy properties. In the present study, a pool of 23 lactic acid bacteria strains was preliminary assayed as monocultures for the fermentation of three ad hoc formulated cereal- (red rice and barley) and pseudocereal (buckwheat) -based substrates. Eight strains with the best performance in terms of acidification rate were selected for the formulation of three multiple strain cultures to be further exploited for the manufacture of laboratory-scale prototypes of fermented beverages. The compositional and microbiological features of the three experimental beverages highlighted their high biological value for further exploitation.

## 1. Introduction

In the last decade, the increase in food intolerances, allergies, and food-based lifestyle choices has dramatically increased the consumer demand for functional foods specifically formulated to ensure a healthy and balanced diet.

Cereal-based beverages undoubtedly belong to this food category, given their beneficial effect on human health [1]. In addition to being sources of carbohydrates, cereals are rich in dietary fiber, micronutrients (B vitamins and tocopherols), phytoestrogens and phenolic compounds [2,3], with acknowledged beneficial effects on human health.

Various cereal-based beverages, such as togwa, boza, taar, amazake and kvass are prepared and consumed worldwide using traditional manufacturing processes and local raw materials [4,5]. For some of these beverages, such as boza or kvass, a spontaneous or even started fermentation process mostly carried out by yeasts and/or lactic acid bacteria converts carbohydrates to alcohol, CO_2_, organic acids and other secondary compounds that deeply affect the sensory properties of the product, while improving its nutritional value, assuring its safety, and prolonging its shelf life [5]. Thanks to their enzymes, microorganisms are also able to degrade antinutrients, such as phytates, as well as to hydrolyze proteins and starch, thus leading to an enhancement of mineral bioavailability and digestibility [5].

Various authors have already suggested how the use of selected lactic acid bacteria with specific flavor- and texture-enhancing properties offers a tool for the innovation and diversification of cereal-based beverages [5].

In the last decade, numerous innovative cereal-based beverages have been developed exploiting selected starter cultures and different cereal grains, including oat (*Avena sativa* L.), emmer (*Triticum dicoccum*) and wheat [5,6,7].

Given the growing interest of both consumers and the food industry for ancient and/or less common grains, the present study was aimed at widening the number of available formulations of innovative beverages characterized by high nutritional value and potential healthy properties. To this end, a pool of 23 lactic acid bacteria strains were preliminary assayed as monocultures for the fermentation of three ad hoc formulated cereal- and pseudocereal-based substrates. Hence, eight strains with the best performance in terms of acidification rate were selected for the formulation of three multiple-strain cultures. These latter were further exploited for the manufacture of laboratory-scale prototypes of fermented beverages using grains with documented valuable biological and/or nutritional traits, including two non-wheat cereals (red rice and barley) and one pseudocereal (buckwheat). Hence, the prototypes were evaluated, immediately after production and/or during their shelf-life under refrigerated conditions, for their chemical (pH, total titratable acidity—TTA), gross compositional/nutritional (moisture, protein, lipids, dry matter, carbohydrates, phytate/total phosphorus, total dietary fiber, ash), and microbiological traits (viable counts of lactic acid bacteria).

## 2. Materials and Methods

### 2.1. Selection of Cereal-Sourced Lactic Acid Bacteria

Twenty-three lactic acid bacteria strains ascribed to *Lacticaseibacillus casei* (basonym *Lactobacillus casei*)/*Lacticaseibacillus paracasei* (basonym *Lactobacillus paracasei*), *Lentilactobacillus parabuchneri* (basonym *Lactobacillus parabuchneri*), *Lentilactobacillus buchneri* (basonym *Lactobacillus buchneri*), *Limosilactobacillus fermentum* (basonym *Lactobacillus fermentum*), *Loigolactobacillus coryniformis* (basonym *Lactobacillus coryniformis*), *Lacticaseibacillus rhamnosus* (basonym *Lactobacillus rhamnosus*), *Pediococcus parvulus*, *Weissella oryzae*, and *Streptococcus thermophilus* (Table 1) were preliminary assayed as monocultures for their ability to ferment three ad hoc formulated cereal- and pseudocereal-based substrates. Among these, twenty strains (labelled BZ) had previously been isolated from commercial Bulgarian boza [8] and three strains were purchased from the International Culture Collection “Deutsche Sammlung von Mikroorganismen und Zellkulturen” (DSM, Braunschweig, Germany). All the strains were maintained at the Culture Collection of the Department of Agricultural, Food and Environmental Sciences (D3A) (Università Politecnica delle Marche, Ancona, Italy) at −80 °C in a mixture of glycerol and de Man, Rogosa and Sharpe (MRS) broth (VWR International, Milan, Italy), at a 2:3 (*v v*^−1^) ratio. Prior to use, the frozen stored cultures were subcultured on MRS agar (VWR) incubated at 30 °C for 48 h, except for *S. thermophilus*, which was incubated at 37 °C.

### 2.2. Selection of Cereal and Pseudocereal Grains with High Biological/Nutritional Value and/or Healthy Traits

For the selection of the cereals and/or pseudocereals to be used for the preparation of the fermentation substrates, a preliminary overview of the scientific studies investigating the biological, nutritional and functional traits of ancient and/or less common grains, never exploited before for the manufacture of fermented beverages was carried out using the PubMed (http://www.ncbi.nlm.nih.gov/pubmed, accessed on 14 February 2021), ScienceDirect (http://www.sciencedirect.com/, accessed on 14 February 2021) and Scopus (https://www.scopus.com/, accessed on 14 February 2021) databases. Based on the available data, three grains were selected, namely red rice, barley, and buckwheat.

### 2.3. Preparation of the ad hoc Formulated Fermentation Substrates

Red rice (RR), barley (B) and buckwheat (Bw) whole grains and maple syrup were purchased from a local retailer (Bio Retail s.r.l., Ancona, Italy). The nutritional label of the three grains and maple syrup is reported in Table 2.

**Figure 1:**
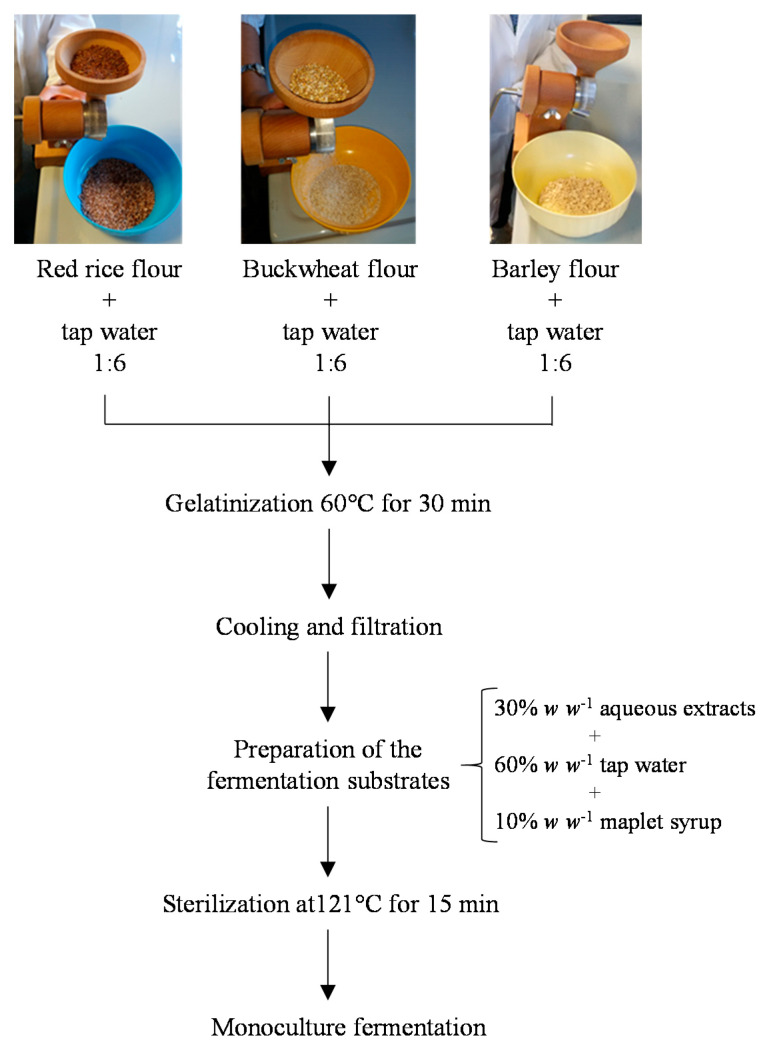
Flow chart of preparation of the ad hoc formulated fermentation substrates.

The grains were separately milled using a manual grinder up until a coarse flour was obtained and used for the preparation of three fermentation substrates, following the flow chart shown in Figure 1.

Briefly, ~100 g of each flour was added to tap water at a 1:6 (*w v*^−1^) ratio, vigorously mixed, and treated at 60 °C for 30 min for gelatinization. Mixtures were cooled at room temperature and filtered using a fine-mesh strainer to eliminate the coarse particulate matter. The obtained percolates were added with tap water and maple syrup at a 3:6:1 (*v v*^−1^) ratio.

For the sole assay of lactic acid bacteria strains as monocultures, the ad hoc formulated substrate was distributed into 10 mL-aliquots in tubes and autoclaved at 121 °C for 15 min for sterilization. After sterilization, a 10 mL aliquot of each coarse flour was analyzed for the content in phytic acid, as detailed in Section 2.7.

### 2.4. Selection of the Multiple-Strain Starters

Lactic acid bacteria were preliminary subcultured on MRS agar (VWR) incubated at 30 °C (except for *S. thermophilus* incubated at 37 °C) to produce visible well separated colonies. Hence, each strain was separately inoculated (in duplicate) into the three ad hoc formulated fermentation substrates prepared with RR, Bw or B, respectively, as detailed in Section 2.3. For each strain, an aliquot of the bacterial biomass was inoculated with a sterile disposable loop (1 μL) under sterile conditions to an initial cell density of ~7 Log colony forming units (cfu) mL^−1^. Cell viability and load were checked, immediately after inoculation, by plate count method on MRS agar (VWR), as detailed in Section 2.9. After 8 and 24 h incubation at 30 °C (except for *S. thermophilus* incubated at 37 °C), aliquots of the fermented substrates were subjected to pH measurement (as detailed in Section 2.6).

Eight strains with the best performance in terms of acidification rate (pH < 5.5 after 8 h fermentation) and acidification extent (pH < 4.8 after 24 h fermentation) were selected for the formulation of three multiple strain starters to be further exploited for the manufacture of laboratory-scale prototypes of fermented beverages with RR, B and Bw, respectively.

### 2.5. Production of Laboratory Scale Prototypes of Fermented Beverages

**Figure 2:**
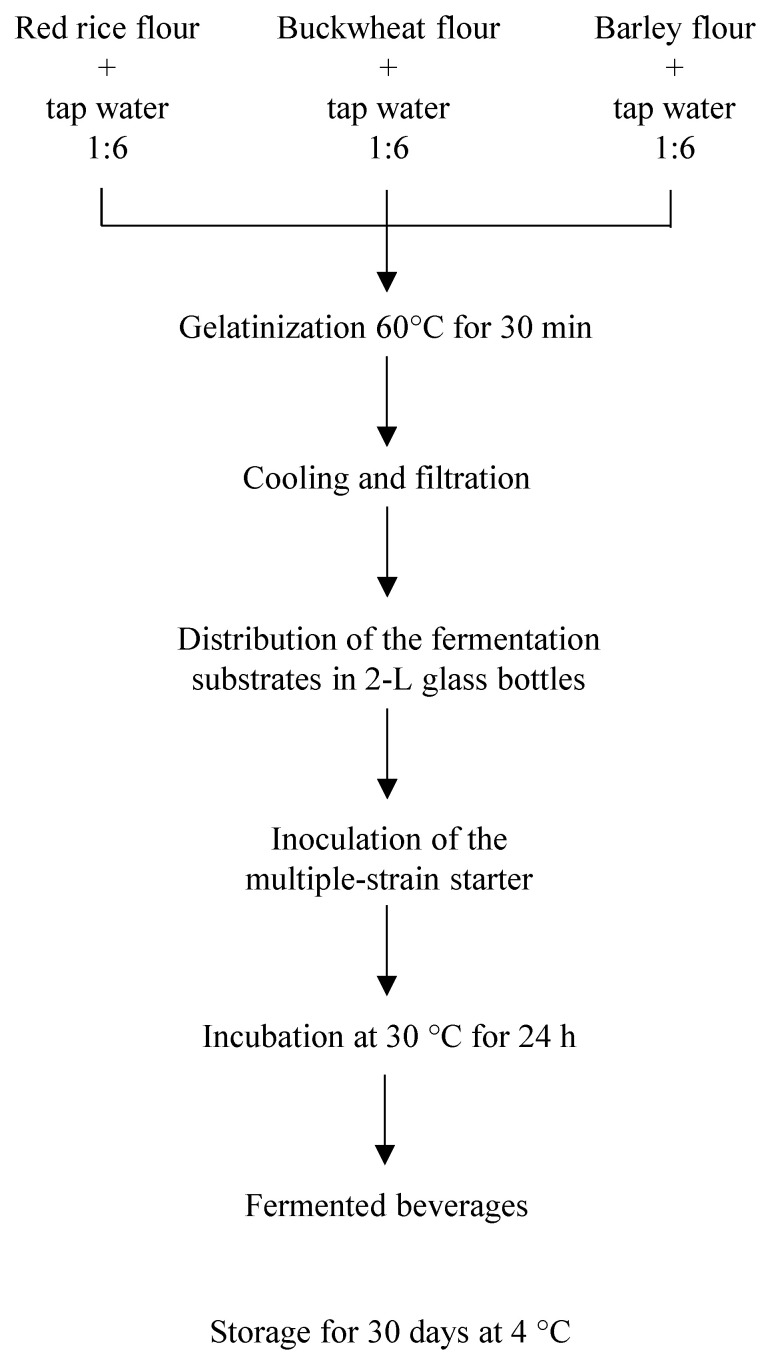
Production of laboratory scale prototypes of fermented beverages.

Approximately 100 g of RR, Bw or B grains were treated as described in Section 2.3 to produce the three fermentation substrates, following the procedure described in Figure 2.

Aliquots (1000 mL) of the three substrates were separately distributed into 2 L sterile glass jars, inoculated (in duplicate) with the specifically formulated multiple strain starter culture under sterile conditions, closed with non-hermetic lids, and incubated at 30 °C for 24 h. For each fermentation substrate, a uninoculated control was also incubated (in duplicate) under the same conditions.

For the preparation of the inoculum, the selected lactic acid bacteria strains were separately subcultured in 10 mL of the opportune fermentation substrates and incubated at 30 °C for 24 h. At the end of the incubation period, the resulting cultures were inoculated into the 2 L glass bottles and vigorously mixed with a sterile tool. Immediately after inoculation, the load of lactic acid bacteria was checked by viable plate counting, as detailed in Section 2.9. Inoculated and uninoculated jars (in duplicate) used as a control were incubated at 30 °C for 24 h. At the end of the fermentation period, they were cooled to +4 °C and maintained under refrigeration for 30 days. Aliquots of the prototypes and the controls sampled under sterile conditions were analyzed for: (i) pH (prior to starter inoculation, after 24 h of fermentation and at regular intervals during their 30 day shelf-life under refrigerated conditions) and total titratable acidity (TTA) (after 24 h of fermentation and at the end of the 30-day shelf life) as detailed in Section 2.6; (ii) phytate/total phosphorus (at the end of the 30-day shelf life) as detailed in Section 2.7.

### 2.6. Determination of pH and Total Titratable Acidity (TTA)

The pH values were determined with a pHmeter (Model 300, Hanna Instruments, Padova, Italy) equipped with a solid electrode (Model HI2031, Hanna Instruments). Three independent measurements were performed for each sample and the results expressed as mean value ± standard deviation.

Total titratable acidity (TTA) was determined as described by Nionelli et al. [7]. Briefly, 10 g of each sample were homogenized with 90 mL of distilled water, and TTA was determined as the volume of 0.1 M NaOH, expressed in mL, used to reach a pH value of 8.3. For each sample, three independent measurements were performed, and the results expressed as mean ± standard deviation.

### 2.7. Determination of Phytate/Total Phosphorus

The quantification of phytic acid (phytate/total phosphorus) was performed using the commercial kit K-PHYT 12/12 (Megazyme, Bray, Ireland) in accordance with the manufacturer’s instructions. For each sample, three independent measurements were performed and the results expressed as mean ± standard deviation.

### 2.8. Determination of Proximate Composition

Dry matter, protein, and ash were determined using the analytical references previously reported by Osimani et al. [11]. The moisture was determined by the gravimetric method (AOAC Official Method 950.46) and total dietary fiber was determined by enzymatic-gravimetric method (AOAC, 991.43), while carbohydrates (CHO) were calculated using the following formula CHO = dry matter − (protein + fat + ash) as previously described [12]. All the analyses were carried out in duplicate, and values were expressed as mean ± standard deviation.

### 2.9. Microbiological Analyses

For the viable counting of lactic acid bacteria, an aliquot of each analyzed sample (1 or 10 g) was accurately homogenized in sterile peptone water (bacteriological peptone, 0.1% *w w*^−1^) at a 1:9 (*w v*^−1^) ratio using a stomacher apparatus for 1 min at 260 rpm. Serial decimal dilutions were prepared with the same diluent and 100 µL of each dilution was inoculated in duplicate by streak-plate technique on MRS agar (VWR) and incubated at 30 °C for 48–72 h in anaerobic jars using AnaeroGen 2.5 L Atmosphere Generation Systems (Thermo Scientific, Massachusetts, USA). Plates showing from 30 to 300 colonies were used for the enumeration. Results were expressed as the mean Log cfu mL^−1^ of three replicates ± standard deviation.

### 2.10. Statistical Analysis

Statistical analyses were performed using the software JMP^®^ Version 11.0.0 (SAS Institute Inc., Cary, NC, USA). The Tukey–Kramer’s Honest Significant Difference (HSD) test (α = 0.05) was carried out to evaluate differences among coarse flours made with RR, BW and B, monocultures, and beverages (prototypes and controls) by one-way analysis of variance (ANOVA).

## 3. Results and Discussion

### 3.1. Selection of the Grains and Formulation of the Fermentation Substrates

In recent decades, there has been an increasing demand for a greater variety of functional foods produced with cereals or pseudocereals possibly enriched with live microorganisms. In this regard, cereals offer many advantages, e.g., they are cultivated and consumed worldwide, and represent rich sources of energy, vitamins, and minerals. Moreover, pseudocereals are characterized by excellent nutritional and biological value defined primarily by the absence of gluten and the high content of components with beneficial effects on human health [13,14]. Finally, the fermentation by lactic acid bacteria of both cereals and pseudocereals can enhance the bioavailability of minerals, digestibility, and sensory properties of the final products. 

Based on the aforementioned premises, three grains with documented functional traits, namely red rice (*Oryza sativa* var. *Indica*, *Tapol*), barley (*Hordeum vulgare* L.), and buckwheat (*Fagopyrum esculentum*) were selected for the development of novel fermented beverages.

According to the available literature, red rice (hereafter referred as RR) is characterized by a high content in phenolic compounds as anthocyanins, phenolic acids and procyanidins [15,16,17], which are known to potentially reduce the oxidation of low-density lipoprotein (LDL) cholesterol and the synthesis of pro-inflammatory compounds and nitric oxide involved in cardiac dysfunctions [15].

Barley (hereafter referred as B) is a cereal with a high concentration of healthy biologically active compounds such as polyphenols, tocopherols, tocotrienols and dietary fibre (*ß*-glucans) [18,19,20]. In more detail, *ß*-glucans can reduce the assimilation of carbohydrates and the production of post-prandial insulin, both in healthy subjects [21] and in subjects with type 2 diabetes or metabolic syndrome [18,22]; they can also reduce plasma cholesterol levels and the risk of heart disease [23,24].

Finally, buckwheat (hereafter referred as Bw) is a pseudocereal with a high content in lysine, dietary fiber, flavonoids (quercetin and rutin), vitamins B1 and B2, phytosterols, soluble carbohydrates and other health beneficial molecules such as D-chiro-inositol and fagopyrins [25,26,27].

To the authors’ knowledge, none of these three grains have ever been exploited for the manufacture of fermented beverages.

For the choice of the C-source to be used for the ad hoc formulation of the fermentation substrate, a search of the available scientific literature was again carried out, having in mind that the substitution of refined sugars with natural sweeteners can be a valid alternative to increase the intake of bio-functional molecules [28]. To date, maple syrup has extensively been studied for its superior sensory profile and its antioxidant, anti-mutagenic and anti-inflammatory properties in respect with other natural sweeteners [29,30]. The maple syrup is also characterized by the presence of other important nutritional/functional components such as minerals, organic acids, amino acids, vitamins, phenolic compounds and phytohormones [31]. Given these data, 10% (*w v*^−1^) maple syrup was added to each fermentation substrates to support microbial growth and metabolic activity.

### 3.2. Formulation of the Multiple Strain Starters

The pool of 23 lactic acid bacteria strains were screened for their ability to ferment the three ad hoc formulated substrates. The results of pH measurement after 8 and 24 h of fermentation in the red rice- barley- and buckwheat-based substrates are shown in Supplementary Table S1. Prior to inoculation, mean pH values attesting at 7.02 ± 0.83, 6.23 ± 0.30 and 7.35 ± 0.24 were measured in the three substrates prepared with RR, B and Bw, respectively. As a general trend, after 8 h of fermentation, the pH was higher than 4.8 for all samples, except for a few isolates, which were characterized by values comprised between 5.15 ± 0.02 and 6.94 ± 0.30 for RR, 5.03 ± 0.32 and 6.13 ± 0.13 for B, and 5.09 ± 0.35 and 7.17 ± 0.16 for Bw; after 24 h, most of the isolates were able to decrease the pH at values below 4.8, irrespective of the substrate considered.

These findings agree well with that reported in the available literature about the suitability of cereals, such as barley and rice, and even pseudocereals to support the growth of pro-technological bacteria [32,33,34,35,36,37] including lactic acid bacteria, which encompasses genera and species naturally contaminating the surface of cereal grains [38]. The ability of candidate starters to grow in cereal-based substrates as well as their viability during storage are important parameters for the development of new fermented cereal-based foods with functional traits. In this regard, growth capability and acidification rate are among the key criteria used for starter selection [39].

The preliminary results overall collected from the screening of the monocultures allowed eight strains to be selected for the formulation of three multiple strain starters, each specific for one non-wheat substrate. The selection was based on pH values < 4.8 reached after 24 h.

The composition of the three multiple strain starters is shown in Table 3.

The eight strains belonged to four species, namely *Lact. casei/Lact. paracasei*, *Loil. coryniformis*, and *Lact. rhamnosus*.

The first two species have an important role in the food industry in relation to their high adaptability to different substrates [40]. *Lact. paracasei* has previously been detected in fruit- or vegetables-based fermented beverages [9,10]. *Lact. casei*, commonly exploited as a starter by the dairy industry, has recently been investigated for its potential application in the production of fermented foods containing cereals, pseudocereals, and legumes [41,42]. In a recent investigation by Puerari et al. [43], this species was found to guide the fermentation process of an unstarted rice-based beverage produced by Umutina Brazilian Amerindians. More recently, Ray et al. [44] reported the presence of *Lact. casei* in fermented Indian foods such as poita bhat, panta bhat and pokhalo produced with rice or a mixture of rice and black beans.

*Lact. rhamnosus* is a further species commercially exploited for the manufacture of fermented foods with healthy properties, given its high potential as a probiotic [45,46]. In recent years, this microorganism has been detected in a rice-based product (rice pudding) [47].

Similarly, to the other three species, even *Loil. coryniformis* has previously been detected in cereal-based beverages, including boza [8,48,49,50], though no application in the food industry as a starter culture has been documented to date, yet.

### 3.3. Manufacture of Prototypes of Fermented Beverages

Three started beverages were produced at laboratory-scale with RR, B and Bw, respectively. The three beverages are depicted in Supplementary Figure S1; they were characterized by a grain-dependent characteristic color, namely light red for RR (due to the occurrence of anthocyanin pigments), light brownish for B, and dark brownish for Bw.

#### 3.3.1. Chemical and Microbiological Analyses

The results of pH measurements carried out at t_0_, after 24 h of fermentation and at regular intervals during the 30 day-storage at +4 °C are shown in Table 4.

At t_0_, initial pH values of 7.23 ± 0.15, 6.95 ± 0.12, and 7.42 ± 0.00 were measured in the substrates made with RR, B and Bw, respectively. A significant pH drop was seen after 24 h of fermentation, with mean values of 4.25 ± 0.05 (RR), 3.95 ± 0.00 (B) and 3.91 ± 0.01 (Bw). Over time, a further progressive pH decrease was seen, with mean values attesting at 4.01 ± 0.03 (RR), 3.51 ± 0.02 (B) and 3.41 ± 0.00 (Bw) detected at the end of the monitoring period. Overall, pH values herein collected were in the range of those previously reported by Coda et al. [51] for a rice-based yogurt-like beverage fermented by *Lactiplantibacillus plantarum* (basonym *Lactobacillus plantarum*) and by Rathore et al. [52] for barley-based beverages fermented by *Lact. plantarum* or *Lactobacillus acidophilus*.

As expected, a decrease in pH corresponded to an increase in TTA. In more detail, in the beverage made with RR, the TTA increased from 0.13 ± 0.01 mL NaOH 0.1 N (0 day) to 1.64 ± 0.07 mL NaOH 0.1 N for the prototypes of fermented beverages made with RR at the end of shelf life; in the beverage made with B, it increased from 2.13 ± 0.05 mL NaOH 0.1 N to 4.95 ± 0.13 mL NaOH 0.1 N, whereas in the beverage containing Bw it passed from 2.65 ± 0.13 mL NaOH 0.1 N (0 day) to 7.60 ± 0.18 mL NaOH 0.1 N (30 days). As it has previously reported, TTA relates to the ‘acid taste’ of a juice or beverage, typically wine [53]. If TTA values herein obtained are compared with those available in the scientific literature, the perceptible acidity of the experimental three beverages was comparable to that of the fermented oat flakes-based beverage produced and analyzed by Nionelli et al. [7].

As far as the viable counts are concerned (Table 4), immediately after inoculation, as expected, lactobacilli reached loads of ~7 Log cfu mL^−1^ in all the three substrates. After 24 h of fermentation, an increase in viable counts from one to two orders of magnitude was seen in the three experimental beverages, with that made with B reaching a mean load as high as 9.30 ± 0.04 Log cfu mL^−1^. Similarly to what was previously demonstrated for other non-wheat grains [7,51,52,54], data herein collected clearly suggested the suitability of RR, B and Bw added with 10% maple syrup to support the growth of lactobacilli. In this regard, the high content of maple syrup in sucrose and, at lesser extent, in small amounts of glucose and fructose has undoubtedly contributed to support the growth of lactic acid bacteria. The loads of lactobacilli found in the three started beverages at the end of the 24 h fermentation were in the range of those reported by various authors for yogurt-like beverages produced with different non-wheat grains [7,51,52], suggesting that the specific strains used as starters were viable, active, and abundant in the final beverages and during their shelf life.

#### 3.3.2. Determination of Phytic Acid/Total Phosphorus

Phytic acid, commonly present in cereals, pseudocereals and legumes is an antinutritional factor which reduces the bioavailability of minerals [55] and negatively affects the absorption of proteins [56] and glucose, thus leading to a decrease in blood glucose response [57]. Fermentation carried out by lactobacilli can lead to a reduction in phytic acid content, thus representing a key aspect in the development of new functional foods [27].

The results of determination of phytic acid/total phosphorus in the coarse flours, experimental and control beverages are shown in Table 5.

No significant differences were observed between the samples analyzed. In RR, B and Bw coarse flours, phytic acid content was 0.314 ± 0.099, 0.407 ± 0.031 and 0.428 ± 0.308 g 100 g^−1^, respectively, whereas in the corresponding experimental beverages, it was 0.084 ± 0.091, 0.196 ± 0.147 g 100 g^−1^, and 0.147 ± 0.010, respectively. Finally, in the controls, it attested at 0.026 ± 0.034, 0.238 ± 0.000 and 0.115 ± 0.025 g 100 g^−1^_,_ respectively.

Overall, the phytic acid content of red rice coarse flour was significantly lower than that reported by Perera et al. [58] for white rice, with values comprised between 8.24 and 17.41 mg g^−1^. Regarding barley, a lower content in respect with values reported by Dai et al. [59] for 100 different barley genotypes, ranging from 3.85 to 9.85 mg g^−1^ was seen. Finally, for buckwheat, data were comparable to those reported by Steadman et al. [60] for buckwheat seeds milled from groats, but significantly lower than those reported by the same authors for bran of buckwheat seeds, with values comprised between 35 and 38 mg g^−1^.

Though lactobacilli are known to potentially produce enzymes able to degrade phytates (phytases) at the low pH values reached during fermentation [61], in the present study, no effect of the multiple strain starter inoculation on the phytate content of the three beverages was seen. This latter finding might be feasibly explained by the lack of phytase activity by the strains used for the formulation of the three multiple strain starters, selected based on their performance as natural acidifiers.

#### 3.3.3. Proximate Composition

The results of proximate composition analysis carried out on the experimental and control beverages are reported in Table 6.

For carbohydrates, significant differences were seen among the three experimental beverages, with values ranging from 8.61 ± 0.08 to 10.31 ± 0.01 g 100 g^−1^ for the started beverages made with Bw and RR, respectively. As expected, no significant differences were seen between the experimental beverages and the corresponding controls.

No significant differences were found between the analyzed samples for fiber concentration, with values comprised between 0.93 ± 0.35 g 100 g^−1^ (control beverage made with Bw) and 1.19 ± 0.11 g 100 g^−1^ (control beverage made with B). Regarding the protein content, by contrast, significant differences were seen among the experimental beverages, with those produced with RR and Bw showing the highest content; significant differences were also found between each experimental beverage and its control. Data overall collected for the protein content of the raw grains agreed well with those reported by Ciesarovà et al. [13] for white rice (6.7–9.4 g 100 g^−1^), barley (10.1–13.4 g 100 g^−1^) and buckwheat (9.7–12.3 g 100 g^−1^). No effect of the multiple strain starter on the protein content of the experimental beverages was seen.

Content of lipids was below the detection limit of 0.07 g 100 g^−1^ in both the experimental and control beverages, irrespective of the grain considered; this finding was quite expected, given the very low lipid content of RR, B and Bw, corresponding to 3.1, 2.3 and 1.7 g 100 g^−1^, respectively. A different picture emerged from the analysis of the dry matter, with the three experimental beverages differing from each other and showing mean values ranging from 9.05 ± 0.07 % to 10.73 ± 0.01 %, for Bw and RR, respectively. In contrast, no significant differences were seen for this component between the experimental beverages and the corresponding controls. Finally, regarding the ash content, no differences emerged between the analyzed samples, with values being comprised between 0.11 ± 0.00 (experimental and control beverage made with B, and control beverage made with Bw) and 0.12 ± 0.00 g 100 g^−1^ (experimental beverage made with RR and Bw), except for the control beverage made with RR, which was characterized by a significantly higher mean content of ash (0.16 ± 0.01 g 100 g^−1^).

## 4. Conclusions

Based on the results overall collected, the following considerations can be made; first, all the three substrates made with RR, B and Bw were effective to support the growth of the inoculated multiple strain starter cultures. Moreover, the new recipes and production processes herein developed allowed three fermented beverages to be manufactured at laboratory-scale; the compositional and microbiological characterization of the three experimental beverages confirmed their high biological value.

All the three beverages showed a high acidity and a high stability of the lactobacilli population during their 30-day storage at +4 °C. Further research is undoubtedly needed to further optimize the production process of the three beverages at the industrial scale and to deepen their nutritional and functional properties. The new beverages produced from uncommon non-wheat grains, including a gluten free pseudocereal, might undoubtedly represent a valid alternative for consumers. Finally, to evaluate the consumer acceptance of the products, a consumer acceptance test carried out on a wide number of subjects is recommended in future.

## Figures and Tables

**Table 1:** The twenty-three lactic acid bacteria strains assayed in the present study for the fermentation of three cereal- and pseudocereal-based substrates.

Strain	Species	Isolation Source	Reference
BZ2	*Limosilactobacillus fermentum* (basonym *Lactobacillus fermentum*)	Bulgarian boza	[8]
BZ5	*Limosilactobacillus fermentum* (basonym *Lactobacillus fermentum*)	Bulgarian boza	[8]
BZ10	*Lacticaseibacillus paracasei* (basonym *Lactobacillus paracasei*)	Bulgarian boza	[8]
BZ21	*Lacticaseibacillus casei* (basonym *Lactobacillus casei*)	Bulgarian boza	[8]
BZ22	*Lacticaseibacillus paracasei* (basonym *Lactobacillus paracasei*)	Bulgarian boza	[8]
BZ26	*Lentilactobacillus parabuchneri* (basonym *Lactobacillus parabuchneri*)	Bulgarian boza	[8]
BZ28	*Lentilactobacillus parabuchneri* (basonym *Lactobacillus parabuchneri*)	Bulgarian boza	[8]
BZ30	*Lentilactobacillus parabuchneri* (basonym *Lactobacillus parabuchneri*)	Bulgarian boza	[8]
BZ31	*Lentilactobacillus parabuchneri* (basonym *Lactobacillus parabuchneri*)	Bulgarian boza	[8]
BZ32	*Lentilactobacillus buchneri* (basonym *Lactobacillus buchneri*)	Bulgarian boza	[8]
BZ33	*Lacticaseibacillus casei* (basonym *Lactobacillus casei*)	Bulgarian boza	[8]
BZ34	*Lacticaseibacillus casei/paracasei* (basonym *Lactobacillus casei/paracasei*)	Bulgarian boza	[8]
BZ35	*Lacticaseibacillus casei* (basonym *Lactobacillus casei*)	Bulgarian boza	[8]
BZ36	*Lentilactobacillus parabuchneri* (basonym *Lactobacillus parabuchneri*)	Bulgarian boza	[8]
BZ37	*Lentilactobacillus parabuchneri* (basonym *Lactobacillus parabuchneri*)	Bulgarian boza	[8]
BZ38	*Lentilactobacillus parabuchneri* (basonym *Lactobacillus parabuchneri*)	Bulgarian boza	[8]
BZ39	*Pediococcus parvulus*	Bulgarian boza	[8]
BZ43	*Loigolactobacillus coryniformis* (basonym *Lactobacillus coryniformis*)	Bulgarian boza	[8]
BZ44	*Loigolactobacillus coryniformis* (basonym *Lactobacillus coryniformis*)	Bulgarian boza	[8]
BZ47	*Lacticaseibacillus casei/paracasei* (basonym *Lactobacillus casei/paracasei*)	Bulgarian boza	[8]
DSM 20021	*Lacticaseibacillus rhamnosus*^T^ (basonym *Lactobacillus rhamnosus*)	Unknown source	
DSM 20617	*Streptococcus thermophiles* ^T^	Pasteurized milk	[9]
DSM 25784	*Weissella oryzae* ^T^	Fermented rice grains	[10]

^T^, type strain.

**Table 2:** Nutritional label of the three commercially available grains and maple syrup purchased from a local retailer (Bio Retail s.r.l.) for the laboratory-scale manufacturing of new fermented beverages.

Nutrient (g/100 g)	Red Rice (RR)	Barley (B)	Buckwheat (Bw)	Maple Syrup
Carbohydrates	68.0	74.0	75.9	90.0 (80.0 sugars)
Proteins	8.7	13.0	9.3	0
Lipids	3.1	2.3	1.7	0
Fibers	7.4	17.0	4.0	0
Salt	0.02	0.03	0.005	0.042
**Caloric Value (Kcal/100 g)**	350	403	362	360

**Table 3:** Multiple-strain starters specifically formulated for the manufacture of laboratory-scale prototypes of the three fermented beverages with red rice (RR), barley (B) and buckwheat (Bw).

Substrate	Multiple-Strain Starter	Strain	Species	pH (8 h)	pH (24 h)
RR	1	BZ22	*Lacticaseibacillus paracasei*	5.65 ± 0.29	4.55 ± 0.13
		BZ33	*Lacticaseibacillus casei*	5.30 ± 0.14	3.97 ± 0.15
		BZ44	*Loigolactobacillus coryniformis*	5.69 ± 0.47	4.40 ± 0.27
		DSM 20021	*Lacticaseibacillus rhamnosus* ^T^	5.70 ± 0.27	3.88 ± 0.10
B	2	BZ33	*Lacticaseibacillus casei*	5.23 ± 0.75	3.82 ± 0.23
		BZ34	*Lacticaseibacillus casei/paracasei*	5.23 ± 0.75	3.82 ± 0.23
		BZ47	*Lacticaseibacillus casei/paracasei*	5.11 ± 0.06	3.83 ± 0.12
		DSM 20021	*Lacticaseibacillus rhamnosus* ^T^	6.13 ± 0.13	3.92 ± 0.07
Bw	3	BZ21	*Lacticaseibacillus casei*	5.42 ± 0.38	3.77 ± 0.05
		BZ22	*Lacticaseibacillus paracasei*	5.35 ± 0.37	4.08 ± 0.08
		BZ35	*Lacticaseibacillus casei*	5.85 ± 0.47	4.13 ± 0.24

^T^, Type strain.

**Table 4:** Viable counts of lactic acid bacteria on Man, Rogosa and Sharpe (MRS) agar incubated at 37 °C, expressed as Log colony forming units (cfu) mL^−1^, and pH values assessed in the laboratory-scale prototypes of fermented beverages made with red rice, barley and buckwheat immediately after inoculation (t_0_), 24 h fermentation, and every 2 days during the 30 day-shelf life under refrigerated conditions (+4 °C) (from t_2_ to t_30_).

t	Red rice (RR)		Barley (B)		Buckwheat (Bw)	
	LAB	pH	LAB	pH	LAB	pH
0	7.14 ± 0.09 ^b^	7.23 ± 0.15 ^a^	6.74 ± 0.03 ^d^	6.95 ± 0.12 ^a^	6.82 ± 0.16 ^b^	7.42 ± 0.00 ^a^
24 h	8.72 ± 0.01 ^a^	4.25 ± 0.05 ^b^	9.30 ± 0.04 ^ab^	3.95 ± 0.00 ^b^	8.65 ± 0.04 ^a^	3.91 ± 0.01 ^b^
2 d	8.72 ± 0.06 ^a^	4.22 ± 0.01 ^bc^	9.32 ± 0.12 ^a^	3.94 ± 0.00 ^bc^	8.82 ± 0.00 ^a^	3.81 ± 0.01 ^b^
4 d	8.59 ± 0.09 ^a^	4.05 ± 0.06 ^bcd^	8.88 ± 0.01 ^c^	3.59 ± 0.03 ^efg^	8.80 ± 0.00 ^a^	3.60 ± 0.01 ^c^
6 d	8.74 ± 0.06 ^a^	4.23 ± 0.06 ^bc^	8.90 ± 0.01 ^c^	3.78 ±0.03 ^cd^	8.73 ± 0.22 ^a^	3.51 ± 0.08 ^cd^
8 d	8.69 ± 0.04 ^a^	4.15 ± 0.08 ^bcd^	8.93 ± 0.01 ^c^	3.59 ± 0.00 ^efg^	8.77 ± 0.10 ^a^	3.47 ± 0.06 ^cd^
10 d	8.67 ± 0.04 ^a^	4.12 ± 0.04 ^bcd^	8.89 ± 0.13 ^c^	3.61 ± 0.01^ef^	8.71 ± 0.45 ^a^	3.45 ± 0.06 ^cd^
12 d	8.70 ± 0.04 ^a^	4.11 ± 0.02 ^bcd^	9.02 ± 0.05 ^abc^	3.68 ± 0.07 ^de^	8.85 ± 0.05 ^a^	3.50 ± 0.03 ^cd^
14 d	8.73 ± 0.01 ^a^	4.11 ± 0.02 ^bcd^	8.96 ± 0.20 ^c^	3.68 ± 0.04 ^de^	8.88 ± 0.07 ^a^	3.50 ± 0.01 ^cd^
16 d	8.66 ± 0.12 ^a^	4.12 ± 0.02 ^bcd^	8.99 ± 0.03 ^bc^	3.59 ± 0.01 ^efg^	8.85 ± 0.03 ^a^	3.39 ± 0.06 ^d^
18 d	8.69 ± 0.07 ^a^	4.13 ± 0.01 ^bcd^	9.03 ± 0.00 ^abc^	3.59 ± 0.07 ^efg^	8.89 ± 0.08 ^a^	3.38 ± 0.03 ^d^
20 d	8.59 ± 0.13 ^a^	4.14 ± 0.04 ^bcd^	8.83 ± 0.12 ^c^	3.60 ± 0.03 ^efg^	9.13 ± 0.50 ^a^	3.46 ± 0.11 ^cd^
22 d	8.64 ± 0.12 ^a^	4.08 ± 0.04 ^cd^	8.91 ± 0.06 ^c^	3.60 ± 0.04 ^efg^	8.91 ± 0.07 ^a^	3.15 ± 0.03 ^e^
24 d	8.65 ± 0.14 ^a^	3.99 ± 0.06 ^bcd^	8.94 ± 0.03 ^c^	3.55 ± 0.01 ^efg^	8.97 ± 0.03 ^a^	3.39 ± 0.01 ^d^
26 d	8.64 ± 0.11 ^a^	4.07 ± 0.04 ^bcd^	8.92 ± 0.05 ^c^	3.44 ± 0.01 ^gh^	8.99 ± 0.24 ^a^	3.43 ± 0.04 ^cd^
28 d	8.52 ± 0.21 ^a^	4.03 ± 0.03 ^bcd^	9.01 ± 0.10 ^abc^	3.38 ± 0.01 ^h^	8.89 ± 0.01 ^a^	3.41 ± 0.04 ^d^
30 d	8.60 ± 0.01 ^a^	4.01 ± 0.03 ^d^	9.11 ± 0.04 ^abc^	3.51 ± 0.02 ^fgh^	9.04 ± 0.01 ^a^	3.41 ± 0.00 ^d^

Means ± standard deviations of triplicate independent experiments are shown. The different letters in the same column indicate significant differences according to the Tukey–Kramer’s (HSD) test (α = 0.05).

**Table 5:** Determination of phytic acid/total phosphorus of coarse flours, prototypes of fermented beverages and control beverage made with red rice (RR), barley (B) and buckwheat (Bw).

Sample		Phytic Acid (g 100 g^−1^)	Total Phosphorus (g 100 g^−1^)
RR	Coarse flours	0.314 ± 0.099 ^a^	0.089 ± 0.028 ^a^
	Prototypes beverage	0.084 ± 0.091 ^a^	0.024 ± 0.026 ^a^
	Control beverages	0.026 ± 0.034 ^a^	0.007 ± 0.010 ^a^
B	Coarse flours	0.407 ± 0.031 ^a^	0.115 ± 0.009 ^a^
	Prototypes beverage	0.196 ± 0.147 ^a^	0.067 ± 0.000 ^a^
	Control beverages	0.238 ± 0.000 ^a^	0.055 ± 0.041 ^a^
Bw	Coarse flours	0.428 ± 0.308 ^a^	0.121 ± 0.087 ^a^
	Prototypes beverage	0.147 ± 0.010 ^a^	0.042 ± 0.003 ^a^
	Control beverages	0.115 ± 0.025 ^a^	0.033 ± 0.007 ^a^

Means ± standard deviations of triplicate independent experiments are shown. Means with the same lowercase superscript letter in the same column are not significantly different according to Tukey–Kramer’s (HSD) test (α = 0.05) between coarse flours, prototypes beverage and control beverages.

**Table 6:** Proximate composition of the prototypes of fermented beverages and control beverage made with red rice (RR), barley (B) and buckwheat (Bw).

Sample		Moisture (%)	Dry Matter (%)	Protein (g 100 g^−1^)	Lipid (g 100 g^−1^)	CHO (g 100 g^−1^)	Fiber (g 100 g^−1^)	Ash (g 100 g^−1^)
RR	Experimental beverage	89.40 ± 0.08 ^d^	10.73 ± 0.01 ^a^	0.28 ± 0.01 ^a^	0.00 ± 0.00 ^a^	10.31 ± 0.01 ^a^	1.15 ± 0.17 ^a^	0.12 ± 0.00 ^b^
	Control beverage	89.27 ± 0.01 ^d^	10.61 ± 0.08 ^a^	0.22 ± 0.00 ^b^	0.00 ± 0.00 ^a^	10.22 ± 0.05 ^a^	1.01 ± 0.46 ^a^	0.16 ± 0.01 ^a^
B	Experimental beverage	90.19 ± 0.01 ^c^	9.81 ± 0.01 ^b^	0.23 ± 0.02 ^b^	0.00 ± 0.00 ^a^	9.28 ± 0.23 ^b^	1.11 ± 0.18 ^a^	0.11 ± 0.00 ^b^
	Control beverage	90.40 ± 0.01 ^bc^	9.60 ± 0.01 ^bc^	0.19 ± 0.03 ^c^	0.00 ± 0.00 ^a^	9.29 ± 0.01 ^b^	1.19 ± 0.11 ^a^	0.11 ± 0.00 ^b^
Bw	Experimental beverage	90.95 ± 0.07 ^a^	9.05 ± 0.07 ^d^	0.28 ± 0.00 ^a^	0.00 ± 0.00 ^a^	8.61 ± 0.08 ^c^	0.94 ± 0.32 ^a^	0.12 ± 0.01 ^b^
	Control beverage	90.74 ± 0.18 ^ab^	9.26 ± 0.18 ^cd^	0.24 ± 0.00 ^b^	0.00 ± 0.00 ^a^	8.87 ± 0.17 ^bc^	0.93 ± 0.35 ^a^	0.11 ± 0.01 ^b^

Means ± standard deviations of duplicate independent experiments are shown. The different letters in the same column indicate significant differences according to Tukey–Kramer’s (HSD) test (α = 0.05). CHO: carbohydrates.

## Data Availability

All data are contained in the text.

## Supplementary Materials

The following are available online at https://www.mdpi.com/2304-8158/10/3/613/s1, Figure S1: Characteristic color of the prototypes of fermented yogurt-like beverages produced with red rice (a); barley (b); and buckwheat (c). Table S1: Results of the pH measurements after 8 and 24 h fermentation of the three substrates made with red rice (RR), barley (B) and buckwheat (Bw) carried out by the 23 strains of lactic acid bacteria.

## References

[1] Coda R., Montemurro M., Rizzello C.G., Shah N.P. (2017). Yogurt-like beverages made with cereals. Yogurt in Health and Disease Prevention.

[2] Katina K., Laitila A., Jovonen R., Liukkonen K.H., Kariluoto S., Piironen V., Landgerg R., Åman P., Poutanen K. (2007). Bran fermentation as a means to enhance technological properties and bioactivity of rye. Food Microbiol..

[3] Basinskienne L., Cizeikiene D., Galanakis C.M. (2020). Cereal-Based Nonalcoholic Beverages. Trends in Nonalcoholic Beverages.

[4] Kandylis P., Pissaridi K., Bekatorou A., Kanellaki M., Koutinas A.A. (2016). Dairy and non-dairy probiotic beverages. Curr. Opin. Food Sci..

[5] Tsafrakidou P., Michaelidou A.-M.G., Biliaderis C. (2020). Fermented Cereal-based Products: Nutritional Aspects, Possible Impact on Gut Microbiota and Health Implications. Foods.

[6] Coda R., Rizzello C.G., Trani A., Gobbetti M. (2011). Manufacture and characterization of functional emmer beverages fermented by selected lactic acid bacteria. Food Microbiol..

[7] Nionelli L., Coda R., José A.C., Poutanen K., Gobbetti M., Rizzello C.G. (2014). Manufacture and characterization of a yogurt-like beverage made with oat flakes fermented by selected lactic acid bacteria. Int. J. Food Microbiol..

[8] Osimani A., Garofalo C., Aquilanti L., Milanović V., Clementi F. (2015). Unpasteurised commercial boza as a source of microbial diversity. Int. J. Food Microbiol..

[9] Marsh A.J., Hill C., Ross R.P., Cotter P.D. (2014). Fermented beverages with health-promoting potential: Past and future perspectives. Trends Food Sci. Technol..

[10] Champagne C.P., Fustier P. (2007). Microencapsulation for the improved delivery of bioactive compounds into foods. Curr. Opin. Biotech..

[11] Osimani A., Garofalo C., Milanović V., Taccari M., Cardinali F., Aquilanti L., Clementi F. (2017). Insight into the proximate composition and microbial diversity of edible insects marketed in the European Union. Eur. Food Res. Technol..

[12] Belleggia L., Ferrocino I., Reale A., Boscaino F., di Renzo T., Corvaglia M.R., Cocolin L., Milanović V., Cardinali F., Garofalo C. (2020). Portuguese cacholeira blood sausage: A first taste of its microbiota and volatile organic compounds. Food Res. Int..

[13] Ciesarová Z., Mikušová L., Osimani M., Kohajdová Z., Karovičová J., Frias J., Martinez-Villaluenga C., Peñas E. (2017). Chapter 17: Nonwheat Cereal-Fermented-Derived Products. Fermented Foods in Health and Disease Prevention.

[14] Barnhoorn R. (2015). Ancient Grains for Modern Times.

[15] Niu Y., Gao B., Slavin M., Zhang X., Yang F., Bao J., Shi H., Xie Z., Yu L. (2013). Phytochemical compositions, and antioxidant and anti-inflammatory properties of twenty-two red rice samples grown in Zhejiang. LWT.

[16] Gunaratne A., Wu K., Li D., Bentota A., Corke H., Cai Y.Z. (2013). Antioxidant activity and nutritional quality of traditional red-grained rice varieties containing proanthocyanidins. Food Chem..

[17] Walter M., Marchesan E., Massoni P.F.S., da Silva P.L., Sartori G.M.S., Ferreira R.B. (2013). Antioxidant properties of rice grains with light brown, red and black pericarp colors and the effect of processing. Food Res. Int..

[18] Belobrajdic D.P., Jobling S.A., Morell M.K., Taketa S., Bird A.R. (2015). Wholegrain barley β-glucan fermentation does not improve glucose tolerance in rats fed a high-fat diet. Nutr. Res..

[19] Lachman J., Hejtmánková A., Orsák M., Popov M., Martinek P. (2018). Tocotrienols and tocopherols in colored-grain whea Ames t, tritordeum and barley. Food Chem..

[20] Martínez M., Motilva M.J., de las Hazas M.C.L., Romero M.P., Vaculova K., Ludwig I.A. (2018). Phytochemical composition and β-glucan content of barley genotypes from two different geographic origins for human health food production. Food Chem..

[21] Harris K.A., Kris-Etherton P.M. (2010). Effects of whole grains on coronary heart disease risk. Curr. Atheroscler. Rep..

[22] Cloetens L., Ulmius M., Johansson-Persson A., Åkesson B., Önning G. (2012). Role of dietary beta-glucans in the prevention of the metabolic syndrome. Nutr. Rev..

[23] Ames N.P., Rhymer C.R. (2008). Issues surrounding health claims for barley. J. Nutr..

[24] De Paula R., Abdel-Aal E.M., Messia M.C., Rabalski I., Marconi E. (2017). Effect of processing on the beta-glucan physicochemical properties in barley and semolina pasta. J. Cereal Sci..

[25] Zhu F. (2016). Buckwheat starch: Structures, properties, and applications. Trends Food Sci. Technol..

[26] Glavač N.K., Stojilkovski K., Kreft S., Park C.H., Kreft I. (2017). Determination of fagopyrins, rutin, and quercetin in Tartary buckwheat products. LWT Food Sci. Technol..

[27] Rollán G.C., Gerez C.L., LeBlanc J.G. (2019). Lactic fermentation as a strategy to improve the nutritional and functional values of pseudocereals. Front. Nutr..

[28] St-Pierre P., Pilon G., Dumais V., Dion C., Dubois M.J., Dubé P., Desjardin Y., Marette A. (2014). Comparative analysis of maple syrup to other natural sweeteners and evaluation of their metabolic responses in healthy rats. J. Funct. Foods.

[29] Singh A.S., Jones A.M.P., Saxena P.K. (2014). Variation and correlation of properties in different grades of maple syrup. Plant. Foods Hum. Nutr..

[30] Nahar P.P., Driscoll M.V., Li L., Slitt A.L., Seeram N.P. (2014). Phenolic mediated anti-inflammatory properties of a maple syrup extract in raw 264.7 murine macrophages. J. Funct. Foods.

[31] Filteau M., Lagacé L., LaPointe G., Roy D. (2012). Maple sap predominant microbial contaminants are correlated with the physicochemical and sensorial properties of maple syrup. Int. J. Food Microbiol..

[32] Angelov A., Gotcheva V., Kuncheva R., Hristozova T. (2006). Development of a new oat-based probiotic drink. Int. J. Food Microbiol..

[33] Mårtensson O., Öste R., Holst O. (2002). The effect of yogurt culture on the survival of probiotic bacteria in oat-based, non-dairy products. Food Res. Int..

[34] Trachoo N., Boudreaux C., Moongngarm A. (2006). Effect of germinated rough rice media on growth of selected probiotic bacteria. Pak. J. Biol. Sci..

[35] Kedia G., Wang R., Patel H., Pandiella S.S. (2007). Used of mixed cultures for the fermentation of cereal-based substrates with potential probiotic properties. Process. Biochem..

[36] Pelikanova J., Liptakova D., Valík L., Stančeková K. (2011). Evaluation of the growth of selected lactobacilli in pseudocereal substrate. Potravinarstvo.

[37] Shori A.B. (2016). Influence of food matrix on the viability of probiotic bacteria: A review based on dairy and non-dairy beverages. Food Biosci..

[38] Guyot J.P. (2012). Cereal-based fermented foods in developing countries: Ancient foods for modern research. Int. J. Food Sci. Technol..

[39] Rather I.A., Seo B., Kumar V.R., Choi U.-H., Lim J., Park Y.-H. (2013). Isolation and characterization of a proteinaceous antifungal compound from *Lactobacillus plantarum* YML007 and its application as a food preservative. Lett. Appl. Microbiol..

[40] Hill D., Sugrue I., Tobin C., Hill C., Stanton C., Ross R.P. (2018). The *Lactobacillus casei* Group: History and Health Related Applications. Front. Microbiol..

[41] Coda R., Rizzello C.G., Gobbetti M. (2010). Use of sourdough fermentation and pseudo-cereals and leguminous flours for the making of a functional bread enriched of gamma-aminobutyric acid (GABA). Int. J. Food Microbiol..

[42] Passerini D., Coddeville M., Le Bourgeois P., Loubière P., Ritzenthaler P., Fontagné- Faucher C., Daveran-Mingot M.L., Cocaign-Bousquet M. (2013). The carbohydrate metabolism signature of *Lactococcus lactis* strain A12 reveals its sourdough ecosystem origin. Appl. Environ. Microbiol..

[43] Puerari C., Magalhães-Guedes K.T., Schwan R.F. (2015). Physicochemical and microbiological characterization of chicha, a rice-based fermented beverage produced by Umutina Brazilian Amerindians. Food Microbiol..

[44] Ray M., Ghosh K., Singh S., Chandra Mondal K. (2016). Folk to functional: An explorative overview of rice based fermented foods and beverages in India. J. Ethn. Foods.

[45] Tamine A.Y., Saarela M., Korslund Sondergaard A., Mistry V.V., Shah N.P., Tamine A.Y. (2005). Production and Maintenance of Viability of Probiotic Microorganisms in Dairy Products. Probiotic Dairy Products.

[46] Shah N.P. (2007). Functional Cultures and Health Benefits. Int. Dairy J..

[47] Williams M., Hekmat S. (2017). *Lactobacillus rhamnosus* GR-1 in Fermented Rice Pudding Supplemented with Short Chain Inulin, Long Chain Inulin, and Oat as a Novel Functional Food. Fermentation.

[48] Todorov S.D. (2010). Diversity of bacteriocinogenic lactic acid bacteria isolated from boza, a cereal-based fermented beverage from Bulgaria. Food Control..

[49] Yeğin S., Üren A. (2008). Biogenic amine content of boza: A traditional cereal-based, fermented Turkish beverage. Food Chem..

[50] Altay F., Karbancioglu-Güler F., Daskaya-Dikmen C., Heperkan D. (2013). A review on traditional Turkish fermented non-alcoholic beverages: Microbiota, fermentation process and quality characteristics. Int. J. Food Microbiol..

[51] Coda R., Lanera A., Trani A., Gobbetti M., di Cagno R. (2012). Yogurt-like beverages made of a mixture of cereals, soy and grape must: Microbiology, texture, nutritional and sensory properties. Int. J. Food Microbiol..

[52] Rathore S., Salmerón I., Pandiella S.S. (2012). Production of potentially probiotic beverages using single and mixed cereal substrates fermented with lactic acid bacteria cultures. Food Microbiol..

[53] Jagtap U., Bapat V.A. (2015). Wines from fruits other than grapes: Current status and future prospectus. Food Biosci..

[54] Matejčeková Z., Liptáková D., Lubomír V. (2017). Functional probiotic products based on fermented buckwheat with *Lactobacillus rhamnosus*. LWT Food Sci. Technol..

[55] Schlemmer U., Frolich W., Prieto R.M., Grases F. (2009). Phytate in foods and significance for humans: Food sources, intake, processing, bioavailability, protective role and analysis. Mol. Nutr. Food Res..

[56] Cheryan M. (1980). Phytic acid interactions in food systems. Crit. Rev. Food Sci. Nutr..

[57] Lee S.H., Park H.J., Chun H.K., Cho S.Y., Cho S.M., Lillehoj H.S. (2006). Dietary phytic acid lowers the blood glucose level in diabetic KK mice. Nutr. Res..

[58] Perera I., Fukushima A., Arai M., Yamada K., Nagasaka S., Seneweera S., Hirotsu N. (2019). Identification of low phytic acid and high Zn bioavailable rice (*Oryza sativa* L.) from 69 accessions of the world rice core collection. J. Cereal Sci..

[59] Dai F., Wang J., Zhang S., Xu Z., Zhang G. (2007). Genotypic and environmental variation in phytic acid content and its relation to protein content and malt quality in barley. Food Chem..

[60] Steadman K.J., Burgoon M.S., Lewis B.A., Edwardson S.E., Obendorf R.L. (2001). Minerals, phytic acid, tannin and rutin in buckwheat seed milling fractions. J. Sci. Food Agric..

[61] Milanović V., Osimani A., Garofalo C., Belleggia L., Maoloni A., Cardinali F., Mozzon M., Foligni R., Aquilanti L., Clementi F. (2020). Selection of cereal-sourced lactic acid bacteria as candidate starters for the baking industry. PLoS ONE.

