# Supplementary material for: Innovative Fermented Beverages Made with Red Rice, Barley, and Buckwheat
Source: Foods. 2021 Mar 13;10(3):613. doi: 10.3390/foods10030613 (PMC8000499; doi:10.3390/foods10030613)
Supplement: Supplementary file 1 [file foods-10-00613-s001.pdf]

Table S1. Results of the pH measurements after 8 and 24 h fermentation of the three substrates made with red rice (RR), barley (B) and buckwheat (B_w) carried out by the 23 strains of lactic acid bacteria.

Strain		**RR**		**B**		**Bw**	
		8h	24h	8h	24h	8h	24h
BZ2	*Limosilactobacillus fermentum*	5.33 ±0.00^{ab}	4.72 ±0.36^{abcd}	4.35 ±0.04^{ab}	4.12 ±0.18^{a}	4.78 ±0.18^{d}	4.43 ±0.17^{cde}
BZ5	*Limosilactobacillus fermentum*	5.37 ±0.02^{ab}	4.68 ±0.15^{abcd}	4.40 ±0.19^{ab}	4.06 ±0.13^{a}	4.46 ±0.38^{d}	4.42 ±0.21^{cde}
BZ10	*Lacticaseibacillus paracasei*	5.74 ±0.64^{ab}	4.39 ±0.12^{abcd}	5.25 ±0.26^{ab}	3.82 ±0.12^{a}	5.55 ±0.54^{abcd}	4.01 ±0.28^{de}
BZ21	*Lacticaseibacillus casei*	5.55 ±0.06^{ab}	4.46 ±0.39^{abcd}	5.54 ±0.06^{ab}	4.33 ±1.04^{a}	5.42 ±0.38^{abcd}	3.77 ±0.05^{e}
BZ22	*Lacticaseibacillus paracasei*	5.65 ±0.29^{ab}	4.55 ±0.13^{abcd}	5.21 ±0.22^{ab}	4.06 ±0.29^{a}	5.35 ±0.37^{abcd}	4.08 ±0.08^{de}
BZ26	*Lentilactobacillus parabuchneri*	5.86 ±0.12^{ab}	4.52 ±0.28^{abcd}	5.05 ±0.43^{ab}	4.22 ±0.33^{a}	7.17 ±0.16^{a}	4.99 ±0.43^{cde}
BZ28	*Lentilactobacillus parabuchneri*	6.12 ±0.63^{ab}	4.84 ±0.14^{abcd}	5.58 ±1.15^{ab}	4.77 ±1.51^{a}	7.05 ±0.18^{ab}	6.70 ±0.30^{ab}
BZ30	*Lentilactobacillus parabuchneri*	6.71 ±0.42^{ab}	5.39 ±0.63^{a}	6.03 ±0.61^{ab}	5.17 ±0.59^{a}	6.31 ±1.37^{abcd}	5.28 ±1.57^{bcd}
BZ31	*Lentilactobacillus parabuchneri*	5.42 ±0.61^{ab}	4.45 ±0.23^{abcd}	4.77 ±0.11^{ab}	3.91 ±0.12^{a}	5.30 ±0.69^{abcd}	4.22 ±0.03^{de}
BZ32	*Lentilactobacillus buchneri*	5.54 ±0.20^{ab}	4.66 ±0.18^{abcd}	5.55 ±0.37^{ab}	4.16 ±0.19^{a}	6.82 ±0.49^{abc}	5.85 ±1.18^{abc}
BZ33	*Lacticaseibacillus casei*	5.30 ±0.14^{b}	3.97 ±0.15^{d}	5.23 ±0.75^{ab}	3.82 ±0.23^{a}	5.53 ±0.08^{abcd}	3.71 ±0.10^{e}
BZ34	*Lacticaseibacillus casei/paracasei*	5.15 ±0.02^{b}	4.11 ±0.04^{cd}	5.17 ±0.33^{ab}	3.69 ±0.15^{a}	5.38 ±0.02^{abcd}	3.73 ±0.11^{e}
BZ35	*Lacticaseibacillus casei*	6.05 ±0.14^{ab}	4.26 ±0.29^{bcd}	5.92 ±0.77^{ab}	4.29 ±0.48^{a}	5.85 ±0.47^{abcd}	4.13 ±0.24^{de}
BZ36	*Lentilactobacillus parabuchneri*	5.17 ±0.66^{b}	4.38 ±0.13^{abcd}	5.25 ±0.61^{ab}	3.98 ±0.12^{a}	5.31 ±0.39^{abcd}	4.33 ±0.08de
BZ37	*Lentilactobacillus parabuchneri*	5.39 ±0.41^{ab}	4.53 ±0.23^{abcd}	5.34 ±0.00^{ab}	4.04 ±0.24^{a}	5.17 ±0.59^{bcd}	4.29 ±0.08^{de}
BZ38	*Lentilactobacillus parabuchneri*	5.28 ±0.61^{b}	4.47 ±0.22^{abcd}	5.03 ±0.32^{ab}	4.08 ±0.21^{a}	5.09 ±0.35^{cd}	4.34 ±0.05^{cde}
BZ39	*Pediococcus parvulus*	6.94 ±0.30^{a}	5.31 ±1.12^{ab}	5.94 ±0.71^{ab}	4.47 ±0.95^{a}	6.94 ±0.14^{abc}	4.46 ±0.21^{de}
BZ43	*Loigolactobacillus coryniformis*	5.45 ±0.02^{ab}	4.61 ±0.14^{abcd}	4.22 ±0.01^{b}	4.01 ±0.18^{a}	4.74 ±0.13^{d}	4.28 ±0.29^{de}
BZ44	*Loigolactobacillus coryniformis*	5.69 ±0.47^{ab}	4.40 ±0.27^{abcd}	4.57 ±0.26^{ab}	3.98 ±0.42^{a}	5.19 ±0.66^{bcd}	4.11 ±0.16^{de}
BZ47	*Lacticaseibacillus casei/paracasei*	5.44 ±0.24^{ab}	4.11 ±0.27^{cd}	5.11 ±0.06^{ab}	3.83 ±0.12^{a}	5.22 ±0.27^{bcd}	3.72 ±0.04^{e}
DSM 20021	*Lacticaseibacillus rhamnosus*	5.70 ±0.27^{ab}	3.88 ±0.10^{d}	6.13 ±0.13^{a}	3.92 ±0.07^{a}	6.93 ±0.16^{abc}	6.97 ±0.23^{a}
DSM 20617	*Streptococcus thermophilus*	5.47 ±0.11^{ab}	5.09 ±0.15^{abc}	5.32 ±0.00^{ab}	4.28 ±0.02^{a}	5.20 ±0.14^{bcd}	4.58 ±0.40^{cde}
DSM 25784	*Weissella oryzae*	6.47 ±0.88^{ab}	5.41 ±0.05^{a}	5.47 ±0.71^{ab}	5.15 ±1.08^{a}	5.89 ±0.52^{abcd}	4.95 ±0.19^{cde}

Means ± standard deviations of the duplicate independent experiments are shown.
The different letters in the same column indicate significant differences according to Tukey–Kramer's (HSD) test ($\alpha = 0.05$).

Figure S1. Characteristic colour of prototypes of fermented yogurt-like beverages produced with red rice (a), barley (b) and buckwheat (c).

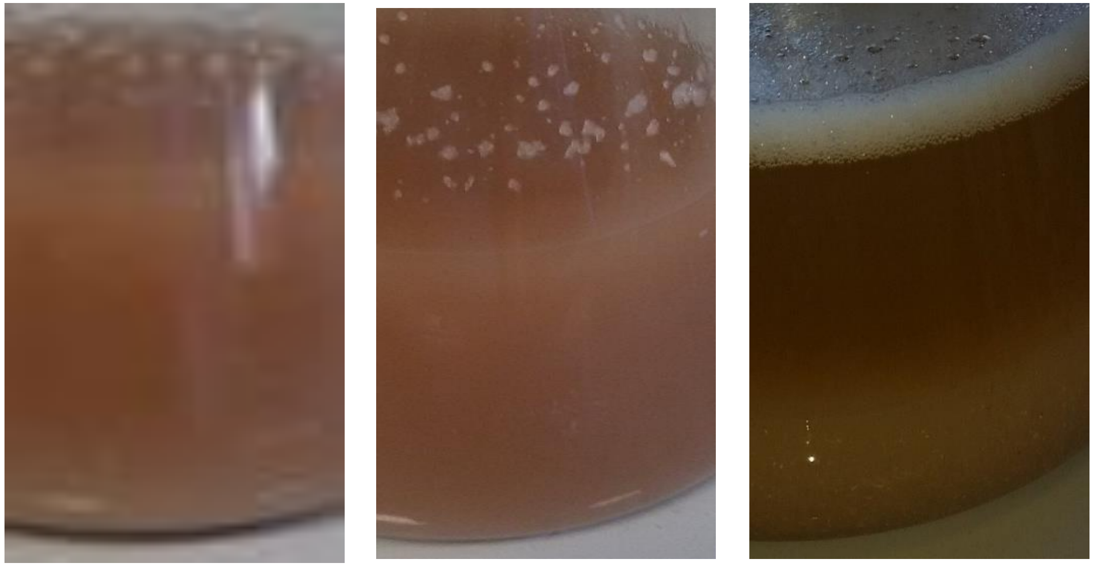

a b c
